# Evaluation of the Impact of Hepatitis B Vaccination in Adults in Jiangsu Province, China

**DOI:** 10.1371/journal.pone.0101501

**Published:** 2014-06-30

**Authors:** Liguo Zhu, Xiangjun Zhai, Yefei Zhu, Weiguo Xu, Changjun Bao, Hong Peng, Qian Bian, Haitao Yang, Hua Wang, Zhibin Hu, Hongbing Shen

**Affiliations:** 1 School of Public Health, Nanjing Medical University, Nanjing, China; 2 Jiangsu Provincial Center for Disease Control and Prevention, Nanjing, China; University of Cincinnati College of Medicine, United States of America

## Abstract

Hepatitis B immunization programs for newborns, children, and adolescents in China have shown remarkable results. To establish whether there would be any benefit in extending the program to cover older individuals, we examined both the epidemiology of hepatitis B virus (HBV) infection and the coverage of hepatitis B vaccinations among adults born before routine vaccinations were implemented. We then evaluated the impact of hepatitis B vaccination in adults aged 20–59 years. A large-scale cross-sectional epidemiological survey of HBV infection was performed in the province of Jiangsu, south-east China, between September 2009 and March 2010. A total of 86,732 adults aged 20–59 years were included, of which 8,615 (9.9%, 95% CI = 9.7–10.1%) were HBsAg sero-positive. Self-reported vaccination status suggested that the coverage was approximately 23.7% (95% CI = 23.4–24.0%). It was shown that higher HBV vaccination coverage was associated with a lower rate of HBsAg seropositivity among adults. There was a negative correlation between hepatitis B vaccination coverage and HBsAg prevalence (correlation coefficient = −0.805, *p* = 0.016), which might demonstrate the combined effects of vaccination and pre-vaccination HBsAg screening. In the unvaccinated group, the HBsAg-positive rate had an obvious upward trend with age growing among 20–39 year-olds (Trend χ^2^ = 22.605, *P*<0.001), while the vaccinated group showed no such trend (Trend χ^2^ = 3.462, *P* = 0.063). Overall, hepatitis B vaccination in adults might reduce the rate of HBsAg positivity. Therefore, routine immunization of adults aged 20–39 years should be seriously considered.

## Introduction

Hepatitis B virus (HBV) infection is a major public health problem in China, a country in which an estimated 95 million people live with chronic HBV infection [1]–[4]. Each year, approximately one-third of the 3 million individuals infected worldwide come from China [5]. Globally, there are ∼600,000 deaths from diseases or liver cancer related to chronic HBV infection, such as cirrhosis, liver failure and hepatocellular carcinoma [6]–[7]. The morbidity, mortality, and medical costs associated with chronic HBV infection are substantial.

In Asia, most of the chronically infected individuals were infected at birth or in early infancy [5], and the significantly increased prevalence rate of anti-HBV core antibody (anti-HBc) with age indicated that horizontal transmission also played an important role in China [2]. Despite the great number of individuals with chronic infection, efforts to control HBV were successful in terms of reducing transmission among the new birth cohorts [8]. China began the routine vaccination of infants against hepatitis B in 1992 [9], and the hepatitis B vaccine was integrated into the national Expanded Program on Immunization (EPI) in 2002 [10]. This was followed in 2009 by a free nationwide catch-up vaccination program (for unvaccinated children and adolescents aged 1–19 years) [11]. Compared with the national serosurvey conducted in 1992, the prevalence of positive hepatitis B surface antigen (HBsAg) among children aged 1–14 years in 2006 was much lower than the same age groups in 1992. In particular, among children <5 years, prevalence was only 1.0%, 90% lower than that in 1992 (9.7%) [12]–[14]. This clearly demonstrates that the introduction of the vaccination program had a remarkable effect to control HBV infection, and that the burden of HBV-related diseases should fall as the vaccinated populations get older [8].

Although newborns, infants, and children below 19 years-of-age are routinely vaccinated against hepatitis B, there is still no suitable adult-based immunization strategy in China. The national serosurvey in 2006 showed that the anti-core positive rate was 47% for those aged 20–59 years. In addition, 40-50% of adults remain susceptible to HBV infection [12]. For an adult, the risk of becoming chronically infected with HBV is less than 5% [15]; however, China is a very populous country in which hepatitis B infection is endemic, so the absolute number of adults at risk of chronic infection is still high. It is recommended that adults at high risk for infection such as injection drug users, sex workers, and health care personnel should be immunized with three doses of HepB vaccine in China [16]. The observed reduction in HBsAg prevalence in North America and Europe is associated with increased hepatitis B vaccine coverage [17]. Therefore, we performed a large-scale epidemiological investigation of adults (aged 20–59 years) in Jiangsu Province, to establish the benefits of extension of the program to cover adults.

## Materials and Methods

### Ethics statement

The study was approved by the Institutional Review Board of Jiangsu Province Center for Disease Control and Prevention. All participants provided written informed consent for blood collection provided and interview.

### Survey population

Three counties selected for this project are Zhangjiagang, Danyang and Taixing, which are located in the east, middle, and north of Jiangsu province, and with income level at high, middle, and low, respectively. Fifty-two villages (18 in Zhangjiagang, 13 in Danyang, and 21 in Taixing) were selected from the total 887 across these three counties based on the systematic random sampling with the average sampling proportion as 1∶17. All the local residents aged 20–59 years who lived in the 52 selected villages for more than 6 months were eligible in the study.

### Investigation method

Before formal investigation, a pilot study including broadcasting and organization, epidemiological questionnaire inquiry and blood collection was conducted in one village from each of the 3 counties in September 2009. We improved the survey procedure after the pilot study. From October 2009 to July 2010, the investigation was formally conducted. In each township hospital, where the selected villages are located, a team of 10–12 investigators (physicians, nurses, and village doctors) was stationed in a central spot for data collection. The investigators were trained by experts comprised with epidemiologists, public health experts from Nanjing Medical University and Jiangsu Province Center for Disease Control and Prevention. A list of all eligible families was generated by visiting all houses before the survey. Village doctors issued a notice of physical examination to each target family, which introduced the survey objective, examination items and attentions. The adults willing to participate arrived at the survey spots in the township hospitals at an appointed time.

Participants were interviewed with a standardized hepatitis B questionnaire, which collect information about the participants’ demographic characteristics, family history of hepatitis B infection, and immunization history. The hepatitis B questionnaire was based on the interview tool used for the 2006 national hepatitis B serological survey. [12] and was modified after the pilot investigation. Vaccination status was based on participant recollection as no immunization records are available for adults. During the interview, the investigators explained the characteristics of HepB vaccine to participants, including the dose and the function. This explanation may help the participants to recall their HBV vaccine status and avoid confusing the screening test with the vaccination. Adults who reported being vaccinated were assumed to have received more than 1 vaccine dose.

A health report including HBsAg test results was sent to participants in a sealed envelope for privacy within two months after examination, and appropriate medical care suggestions were provided according to their infection status. Data were stored in a secure place and were analysed anonymously.

### Blood collection, processing, and laboratory testing

Blood samples (5 ml) were collected with disposable vacuum syringes. All serum specimens were separated on the same day of blood collection and stored at −20°C until testing for HBsAg in local Centers for Disease Control and Prevention laboratories using a detailed laboratory-testing protocol that had been established. HBsAg was measured by an enzyme-linked immunosorbent assay (ELISA) with the use of a commercial kit (Shanghai Ke Hua Bioengineering Co., Ltd., Shanghai, China). The lower detection limit of the HBsAg ELISA method was 0.2 IU/ml. In the pilot survey, 1936 serum samples were randomly selected and tested using the ELISA reagents and Abbott Chemiluminesent Microparticle ImmunoAssay (CMIA) reagents simultaneously. It was found that the testing results between the two methods in detecting HBsAg were consistent (Kappa value = 0.9373) (unpublished data).

### Statistical analysis

The overall prevalence of HBsAg sero-positivity and self-reported vaccination coverage among adults, together with their 95% confidence intervals (CI), was calculated by Stata Version 10.0 software (Stata, College Sation, TX). Standardized prevalence of HBsAg sero-positivity in the three counties was calculated according to the distributions of the sex and age of the adults (20–59) in Jiangsu in 2010. The formula of standardization method was as follows: 
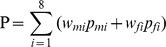



Where, W_mi_ and W_fi_ represent the proportion of the male and female adults in the whole province in age group i, and P_mi_ and P_fi_ represent the prevalence of HBsAg sero-positivity of male and female adults in age group i in this survey.

Association between participants’ demographics, immunization history and prevalence of HBsAg was analyzed using the χ^2^ test and correlation analysis by SPSS 13.0 software (SPSS, Inc., Chicago, IL, USA). The trend of HBsAg sero-positive rates with age was also analyzed using trend χ^2^ tests. Meanwhile, multivariate logistic regression analysis was used to identify factors associated with HBsAg-positive rates among the study population. A two-sided *p-*value <0.05 was considered statistically significant.

## Results

### Characteristics of the study population

A total of 92,641 adults met the study criteria and 86,732 agreed to participate in the study to donate their blood samples and undergo HBsAg testing, yielding a response rate as 93.6%. Characteristics of the study subjects were shown by Table 1. 14.1% were aged 20–29 years, 23.7% were aged 30–39 years, 34.3% were aged 40–49 years, and 27.9% were aged 50–59 years. The male-to-female ratio was 0.73∶1. Geographically, 33.3% of the participants were from Zhangjiagang, 30.5% were from Danyang, and 36.2% were from Taixing. Overall, 23.7% (95% CI = 23.4–24.0%) self-reported to have been immunized against hepatitis B, 58.8% (95% CI = 58.5–59.1%) reported no vaccination history, and 17.5% (95% CI = 17.3–17.8%) reported unknown.

**Table 1:** Characteristics of the 86,732 study participants.

Characteristic	Frequency (No.)	Proportion (%)
Age (years)		
20∼	5,917	6.8
25∼	6,316	7.3
30∼	9,106	10.5
35∼	11,460	13.2
40∼	15,371	17.7
45∼	14,397	16.6
50∼	10,741	12.4
55∼	13,424	15.5
Gender		
Male	36,626	42.2
Female	50,106	57.8
Area		
Zhangjiagang	28,881	33.3
Danyang	26,466	30.5
Taixing	31,385	36.2
Self-reported hepatitis B vaccine uptake		
No	50,991	58.8
Yes	20,557	23.7
Unknown	15,184	17.5

### HBsAg-positivity rates

The overall prevalence of HBsAg seropositivity was 9.9%(95% CI = 9.7–10.1%). The standardized prevalence was 9.8%, adjusted to represent the population of Jiangsu province (20–59 years) in 2010. The prevalence of HBsAg positivity by different characteristics was shown in Table 2. Overall, the HBsAg-positive prevalence increased steadily from 6.6% among adults age 20–24 years, to 11.6% among persons age 35–39 years; however, it declined slightly among those aged 40–59 years.

**Table 2:** HBsAg-positive rate according to socio-demographic characteristics.

Characteristic	Sample tested	HBsAg sero-positive (n)	Prevalence (%)	95%CI	OR (95% CI)	MLR-OR*
Age (years)						
20∼	5,917	391	6.6	6.0–7.3	1.0 (reference)	1.0 (reference)
25∼	6,316	545	8.6	7.9–9.3	1.34 (1.17–1.53)	1.28 (1.12–1.47)
30∼	9,106	972	10.7	10.0–11.3	1.69 (1.50–1.91)	1.46 (1.29–1.66)
35∼	11,460	1,331	11.6	11.0–12.2	1.86 (1.65–2.09)	1.59 (1.41–1.79)
40∼	15,371	1,576	10.3	9.8–10.7	1.62 (1.44–1.81)	1.44 (1.28–1.63)
45∼	14,397	1,421	9.9	9.4–10.4	1.55 (1.38–1.74)	1.42 (1.26–1.61)
50∼	10,741	1,106	10.3	9.7–10.9	1.62 (1.44–1.83)	1.43 (1.26–1.61)
55∼	13,424	1,273	9.5	9.0–10.0	1.48 (1.32–1.67)	1.32 (1.17–1.49)
Gender						
Male	36,626	4,333	11.8	11.5–12.2	1.0 (reference)	1.0 (reference)
Female	50,106	4,282	8.6	8.3–8.8	0.70 (0.67–0.73)	0.68 (0.65–0.71)
Area						
Zhangjiagang	28,881	1,864	6.5	6.2–6.7	1.0 (reference)	1.0 (reference)
Danyang	26,466	2,466	9.3	9.0–9.7	1.49 (1.40–1.59)	1.38 (1.29–1.47)
Taixing	31,385	4,285	13.7	13.3–14.0	2.29 (2.17–2.43)	2.21 (2.08–2.34)
Self-reported hepatitis B vaccine uptake						
No	50,991	5956	11.7	11.4–12.0	1.0 (reference)	1.0 (reference)
Yes	20,557	1258	6.1	5.8–6.5	0.49 (0.46–0.53)	0.54 (0.51–0.58)
Unknown	15,184	1401	9.2	8.8–9.7	0.77 (0.72–0.82)	0.88 (0.83–0.94)

*Adjusted for age, gender, geographic area and self-reported hepatitis B vaccination uptake.

The HBsAg-positive rates of participants in Zhangjiagang, Danyang, and Taixing were 6.5%, 9.3% and 13.7%, respectively, while the standardized prevalence in the three counties were 6.1%, 9.3% and 13.4%, respectively. The male and female rates of the HBsAg-positive in all three counties were 11.8% and 8.6%.

### Self-reported vaccination coverage according to age and region

The self-reported coverage of hepatitis B vaccination was shown in Table 3. Overall, self-reported vaccination coverage in the three counties was approximately 23.7% (95% CI = 23.4–24.0%), with greatest rate in Zhangjiagang (33.6%) and lowest rate in Danyang (10.9%). Among those aged 20–59 years, coverage fell from 60.3% to 16.2% as age increased. The level of hepatitis B vaccination coverage in the three areas varied significantly with age (*p*-value <0.001). The vaccination coverage among individuals aged 35–59 years was higher in Zhangjiagang (31.2%) than that in Danyang (6.6%) and that in Taixing (13.1%). The self-reported rate of hepatitis B vaccination among individuals aged 20–34 years was higher in Taixing (49.5%) than the self-reported rates in Zhangjiagang (41.8%) and Danyang (32.2%).

**Table 3:** Overall coverage and number of participants who self-reported hepatitis B vaccination according to age.

Characteristic	Zhangjiagang	Danyang	Taixing	Total	*P*-value*
Total	9,692 (33.6)	2,880 (10.9)	7,985 (25.4)	20,557 (23.7)	< 0.001
Age (years)					
20∼	963 (55.9)	581 (48.3)	2,022 (67.6)	3,566 (60.3)	< 0.001
25∼	794 (41.0)	414 (32.8)	1,731 (55.6)	2,939 (46.5)	< 0.001
30∼	860 (33.1)	429 (21.9)	1,524 (33.5)	2,813 (30.9)	< 0.001
35∼	951 (28.8)	447 (13.7)	1,016 (20.8)	2,414 (21.1)	< 0.001
40∼	1,548 (28.0)	369 (7.9)	747 (14.4)	2,664 (17.3)	< 0.001
45∼	1,562 (29.6)	339 (6.4)	446 (11.6)	2,347 (16.3)	< 0.001
50∼	1,250 (33.0)	127 (3.4)	269 (8.3)	1,646 (15.3)	< 0.001
55∼	1,764 (37.3)	174 (3.4)	230 (6.4)	2,168 (16.2)	< 0.001

* *P* value is the result of comparing among the three counties.

### Relationship between self-reported rates of vaccination and HBsAg seropositivity

Table 4 showed that the vaccinated group had a significantly lower prevalence of HBsAg positivity compared with those of unvaccinated group and unknown group (*P*<0.001), which was consistent across different age groups. The prevalence of HBsAg-positive among vaccinated adults was no more than 7.4% in each age group, however, the HBsAg-positive rates for unvaccinated adults was 15.8% in Taixing, 9.4% in Danyang, and 7.9% in Zhangjiagang. In multivariate logistic regression analysis, an independent predictor for the HBsAg-positive rate was hepatitis B vaccination (adjusted odds ratio (AOR)  = 0.54; 95% CI = 0.51–0.58), controlling for age, gender and geographic area (Table 2). There was a negative correlation between hepatitis B vaccination coverage and HBsAg prevalence rate (correlation coefficient = −0.805, *p* = 0.016).

**Table 4:** Relationship between hepatitis B vaccination and HBsAg seropositivity.

Characteristic	Vaccinated		Not Vaccinated		Unknown		*P*-value*
	Investigated	Prevalence (%)	Investigated	Prevalence (%)	Investigated	Prevalence (%)	
Total	20,557	6.1	50,991	11.7	15,184	9.2	< 0.001
Age (years)							
20∼	3,566	5.7	1,696	8.8	655	5.8	< 0.001
25∼	2,939	6.6	2,371	11.2	1,006	8.6	< 0.001
30∼	2,813	6.9	4,677	13.0	1,616	10.3	< 0.001
35∼	2,414	7.4	6,903	13.7	2,143	9.7	< 0.001
40∼	2,664	5.8	9,879	11.9	2,828	8.9	< 0.001
45∼	2,347	5.2	9,300	11.1	2,750	9.6	< 0.001
50∼	1,646	5.9	7,222	11.5	1,873	9.6	< 0.001
55∼	2,168	5.2	8,943	10.7	2,313	8.9	< 0.001
Gender							
Male	9,052	7.0	21,070	14.1	6,504	11.0	< 0.001
Female	11,505	5.4	29,921	9.9	8,680	7.9	< 0.001
Area							
Zhangjiagang	9,692	4.4	15,666	7.9	3,523	5.5	< 0.001
Danyang	2,880	6.7	13,732	9.4	9,854	9.9	< 0.001
Taixing	7,985	8.0	21,593	15.8	1,807	12.6	< 0.001

* *P* value is the result of comparing among all three vaccination status.

**Figure 1:**
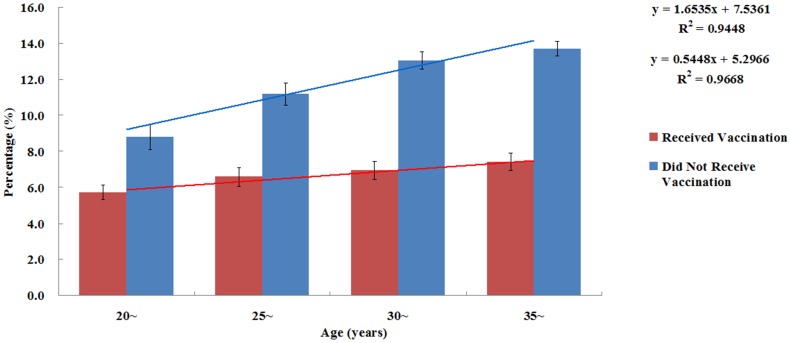
Percentages of infections broken down by immunization in the different age-classes. Differences in prevalence for the unvaccinated population were highly significant in each age group under age 39 years (Trend χ2 = 22.605, P<0.001), after which they did not vary significantly. In the vaccinated population, the HBsAg positive rates in different age groups were all around 6.0%, and the trend of HBsAg-positive prevalence changed by age was different to that in the unvaccinated population (Trend χ2 = 3.462, P = 0.063).

The rates of HBsAg positivity by age among vaccinated and unvaccinated adults were shown in Figure 1. Differences in prevalence for the unvaccinated population were greatly significant in each age group under age 39 years (Trend χ^2^ = 22.605, *P*<0.001), but there is no difference for the age group greater than 39. The rates of HBsAg positivity in each age group under age 39 years in the vaccinated population was all around 6.0%, and the trend of HBsAg-positive prevalence changed by age was different to that in the unvaccinated population (Trend χ^2^ = 3.462, *P* = 0.063).

## Discussion

The overall HBsAg-positive rate among adults in the three counties was 9.9%, similar to the standardized prevalence (9.8%) but slightly higher than that reported in the 2006 national epidemiology survey (9.0%) [12]. The HBsAg-positive rates differed across Zhangjiagang, Danyang, and Taixing, which were 6.5%, 9.3% and 13.7%, respectively. This may be related to endemicity carried down from one generation to the next by vertical and horizontal transmission [5]. Previous studies indicated that the HBsAg-positive prevalence among adults aged 20–49 years in Taixing was 15.5% in 2001 [18], and the prevalence among adults >15 years-of-age in Zhangjiagang was 11.1% in 2006 [19]. Taixing is located in the northern part of Jiangsu Province with a less developed economy and poorer public healthcare capacity, which may also be related to higher hepatitis B prevalence [2], [20].

Overall, HBsAg positivity tended to increase with age, before declining slightly in those aged 40–59 years. The HBsAg positive rate among those aged 20–24 was 6.6%, much lower than that in older age groups, and this may be partly because of the effect of vaccination in adults. Since the earliest use of HepB vaccine in Jiangsu province was in 1987 [19], the 20–24 age group had more opportunity to be vaccinated at birth or in early childhood and had more benefits to avoid chronic HBV infection. The increase of HBsAg positive rate in the population aged 20–39 years, especially 25–39 years, who have had no chance to be vaccinated at birth or in early childhood, can also be caused by an increase in social activities associated with HBV transmission such as sexual activity and occupational exposure, illustrating that the population in this group has an accumulated risk of hepatitis B infection.

Data from the current survey showed that HBsAg positivity was higher in males than in females, which is consistent with previous studies [2], [21]. It may infer that sex hormones and differences in lifestyle may be related to disparity in status of hepatitis B infection [22]–[24]. The prevalence among child bearing age women in these three counties was still high, so it is important to ensure that newborns receive a dose of vaccine within 24 hours of life and hepatitis B immunoglobulin (HBIG) if the mother is HBsAg positive to prevent perinatal transmission or mother to child transmission [5]. This passive-active immunization strategy is now implemented in China.

A previous study indicates that 13.8% of participants aged 15–59 years reported receiving a hepatitis vaccination in 2006 [10]. The increase in hepatitis B vaccination coverage (23.7%) among adults identified in the present study demonstrates substantial progress. The adult hepatitis B vaccination rate in Zhangjiagang was greater than that in the other two counties, particularly in those aged >35 years. A local health policy in Zhangjiagang made the hepatitis B vaccine free of charge for adults since 2007, resulting in more than 200,000 people being vaccinated (data unpublished). The vaccination coverage was the highest in Zhangjiagang because of this free immunization policy and local healthcare promotion for all adults about hepatitis B vaccinations [25]. It is noteworthy that in Taixing, those aged 20–34 years reported the highest rate of hepatitis B vaccination, indicating that the difference between the three counties in terms of HBsAg-prevalence rates for this particular age group was smaller than for the other age groups. Thus, it appears that the rate of HBsAg prevalence in Taixing will fall.

The HBsAg-positive rate was significantly lower in participants who had been vaccinated against hepatitis B than in those who had not been vaccinated (or in those with unknown vaccination status), which is consistent with the 2006 national HBV epidemiology survey results [10]. Multivariate logistic regression analysis revealed a strong negative association between hepatitis B vaccination and serum HBsAg positivity, but this may be because of the combined effects of vaccination and pre-vaccination HBsAg screening. With screening, the HBsAg positive people would not receive vaccination, while those HBsAg negative would receive hepatitis B vaccination. Therefore, adults not vaccinated would have a higher HBsAg-positive rate than the vaccinated. Moreover, our research demonstrated the effects of the vaccination in adults, in addition to the screening effect presented in all age groups, that is to say, if the vaccination had no effects, the trend of HBsAg-positive prevalence changed by age in vaccinated and not vaccinated groups would be similar. However, our research data showed the HBsAg-positive rate had an obvious upward trend among 20–39 year-olds in the unvaccinated group, while the vaccinated group showed no such trend. It showed that hepatitis B vaccine immunization among 20–39 year-olds can slow the rise of the adult’s hepatitis B infection.

We found that the HBsAg-positive rate among adults who self-reported vaccinated was 6.1%, indicating that they might have already been infected with HBV by the time they were vaccinated. An alternative explanation may be that some individuals incorrectly assumed their vaccination status or were part of the unlucky ∼5% of the population not protected by the vaccine. According to the national sero-survey in 2006, the anti-HBc positive rate was 47% for adults aged 20–59 years [12]. We suggest that a testing of HBsAg and anti-HBc for adults before vaccination might be cost-efficient, and routine screening among adults at high risk should be promoted in those areas with high prevalence.

This study has several limitations that should be considered. First, we estimated the vaccination coverage rate from self-reported questionnaires, so the results may be subject to recall bias. The second limitation is that we were not aware of the actual doses of hepatitis B vaccine received. It is recommended that adults should be immunized with three doses of HepB vaccine, but in the vaccinated population, percentage of the fulfillment of recommended doses was usually less than 100% [25], which may reduce the immune efficiency, so the effects of HepB vaccination for adults may be underestimated in this study. Third, the three counties were not randomly selected and may not fully represent the Province. The provincial estimate may over or under estimate the true prevalence for the province. In addition, 6.4% of the interviewees, most of which were male and young (aged 20–29 years), were not present during the survey.

In conclusion, adults that were not reached by the national hepatitis B vaccination campaign showed a high rate of HBsAg positivity. The results of the present study suggest that the age range for hepatitis B vaccination in China should be extended to include those adults aged 20–39 years. Healthcare providers are encouraged to adopt strategies that ensure adults at risk for HBV infection have access to hepatitis B vaccines. Hepatitis B vaccinations should be covered by the national medical insurance system, thereby providing greater access to vaccination and increasing overall coverage. The findings reported herein may help to formulate future policies regarding hepatitis B immunization among adults to improve HBV control and prevention.
